# PER1 suppresses glycolysis and cell proliferation in oral squamous cell carcinoma via the PER1/RACK1/PI3K signaling complex

**DOI:** 10.1038/s41419-021-03563-5

**Published:** 2021-03-15

**Authors:** Xiaobao Gong, Hong Tang, Kai Yang

**Affiliations:** Department of Oral and Maxillofacial Surgery, The First Affiliated Hospital of Chongqing Medical University, No. 1, Youyi Road, Yuzhong District, 400016 Chongqing, China

**Keywords:** Oral cancer, Cell growth, RNAi

## Abstract

There is increasing evidence that the core clock gene Period 1 (PER1) plays important roles in the formation of various tumors. However, the biological functions and mechanism of *PER1* in promoting tumor progression remain largely unknown. Here, we discovered that PER1 was markedly downregulated in oral squamous cell carcinoma (OSCC). Then, OSCC cell lines with stable overexpression, knockdown, and mutation of PER1 were established. We found that PER1 overexpression significantly inhibited glycolysis, glucose uptake, proliferation, and the PI3K/AKT pathway in OSCC cells. The opposite effects were observed in PER1-knockdown OSCC cells. After treatment of PER1-overexpressing OSCC cells with an AKT activator or treatment of PER1-knockdown OSCC cells with an AKT inhibitor, glycolysis, glucose uptake, and proliferation were markedly rescued. In addition, after treatment of PER1-knockdown OSCC cells with a glycolysis inhibitor, the increase in cell proliferation was significantly reversed. Further, coimmunoprecipitation (Co-IP) and cycloheximide (CHX) chase experiment demonstrated that PER1 can bind with RACK1 and PI3K to form the PER1/RACK1/PI3K complex in OSCC cells. In PER1-overexpressing OSCC cells, the abundance of the PER1/RACK1/PI3K complex was significantly increased, the half-life of PI3K was markedly decreased, and glycolysis, proliferation, and the PI3K/AKT pathway were significantly inhibited. However, these effects were markedly reversed in PER1-mutant OSCC cells. In vivo tumorigenicity assays confirmed that PER1 overexpression inhibited tumor growth while suppressing glycolysis, proliferation, and the PI3K/AKT pathway. Collectively, this study generated the novel findings that PER1 suppresses OSCC progression by inhibiting glycolysis-mediated cell proliferation via the formation of the PER1/RACK1/PI3K complex to regulate the stability of PI3K and the PI3K/AKT pathway-dependent manner and that PER1 could potentially be a valuable therapeutic target in OSCC.

## Introduction

Oral squamous cell carcinoma (OSCC) accounts for approximately 90% of oral cancer cases^1^ and is one of the most common malignant tumors of the head and neck; moreover, its incidence rate is increasing annually^2,3^. Although great progress has been made in surgery, radiotherapy, chemotherapy, and other technologies to date, and various new treatment methods are emerging^4^, the 5-year overall survival rate of patients with OSCC has remained at approximately 50% over the past 30 years and has not improved significantly^5^. Therefore, in-depth study of the mechanism by which OSCC occurs is highly important to find new therapeutic targets in order to improve the survival rate of patients with OSCC.

Circadian clock genes exist in almost all cells of the human body^6,7^ and play regulatory roles in many important cell biological processes^8–10^. Period 1 (*PER1*) is one of the core circadian clock components^7,11^, and it can regulate many important physiological activities, such as the cell cycle and apoptosis^9,12^. Current studies have shown that abnormal expression of *PER1* is closely related to the occurrence and development of many kinds of cancers, such as gastric cancer and non-small cell lung cancer (NSCLC)^13,14^. We previously found that the expression of *PER1* was decreased in OSCC and was significantly correlated with clinical stage and survival time^15,16^. The above studies indicate that *PER1* is an important tumor suppressor; however, the underlying mechanism is still unclear. Therefore, it is possible to obtain valuable findings through an in-depth study of *PER1*.

It has been confirmed that the growth of normal cells mainly depends on oxidative phosphorylation using glucose for energy, while energy for the growth of tumor cells is obtained mainly from glucose via glycolysis; aerobic glycolysis is one of the most basic biochemical characteristics of cancer cells^17,18^. The search for therapeutic targets aimed at glycolysis of cancer cells has shown great prospects for the development of cancer treatments^19–21^; however, it is currently unclear whether *PER1* can regulate glycolysis in cancer cells. Current studies have demonstrated that the phosphoinositide-3 kinase (PI3K)/AKT pathway is an important pathway in the regulation of cell glycolysis and proliferation^22,23^. Our previous study demonstrated that the p-AKT level and cell proliferation increased significantly after knockdown of *PER1* in OSCC cells^16^. Therefore, we speculated that *PER1* may regulate glycolysis through the PI3K/AKT pathway, thus affecting the occurrence and development of OSCC. A further important unknown is the possible mechanism by which *PER1* regulates the PI3K/AKT pathway. Current studies have confirmed that RACK1 (receptor for activated C kinase 1) is a scaffold protein, which is upregulated in many human cancers, including OSCC^24–26^. Hu et al. reported that PER1 bound to the RACK1 protein through its PAS domain to form the PER1/RACK1 complex in human suprachiasmatic nucleus (SCN) cells^27^. Cao et al. found that RACK1 bound with PI3K to form the RACK1/PI3K complex in human breast cancer cells^28^. It is not clear whether the PER1/RACK1/PI3K complex exists in cells. However, from the above findings, we can infer that, in OSCC cells, PER1 may bind with RACK1 and PI3K to form the PER1/RACK1/PI3K complex, which can mediate a change in PI3K protein stability and thus regulate the PI3K/AKT pathway and glycolysis.

In this study, we established OSCC cell lines with stable overexpression, knockdown, and mutation of *PER1* and performed functional rescue experiments by adding an AKT activator, AKT inhibitor, or glycolysis inhibitor. The aim of this study was to demonstrate that, in OSCC cells, PER1 is dependent on the formation of the PER1/RACK1/PI3K complex to regulate PI3K protein stability and the PI3K/AKT pathway and regulates glycolysis in a manner dependent on the PI3K/AKT pathway; in turn, its subsequent regulation of cell proliferation depends on glycolysis. Furthermore, we investigated whether overexpression of PER1 significantly inhibited the growth of OSCC tumors, the PI3K/AKT pathway, glycolysis, and proliferation through tumorigenesis experiments in vivo. This study is of great significance for elucidating the biological function of the circadian clock gene *PER1* and its tumor-inhibition mechanism in OSCC and provides a basis for further study of PER1 as a potential target for the treatment of OSCC.

## Results

### The expression of PER1 was low in OSCC cells

**Fig. 1:**
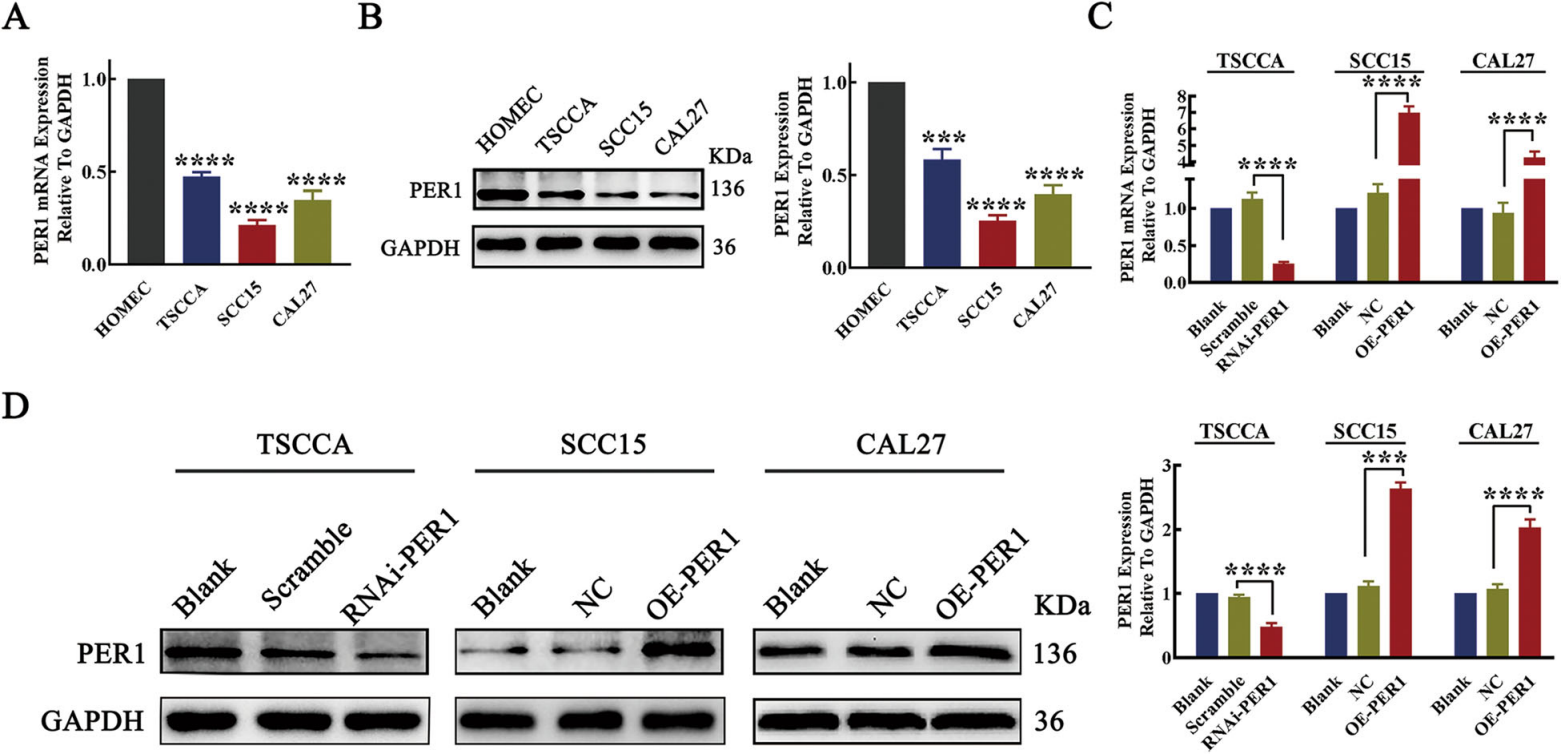
The expression of PER1 was low in OSCC cells. **A** RT-qPCR results showed that the expression of PER1 mRNA in TSCCA, SCC15, and CAL27 cells was significantly lower than that in HOMEC cells; **B** western blot results showed that the expression of PER1 protein in TSCCA, SCC15, and CAL27 cells was significantly lower than that in HOMEC cells; **C**, **D** RT-qPCR and western blot results showed that the mRNA and protein expression levels of PER1 in RNAi-PER1-TSCCA cells were significantly decreased, while those in OE-PER1-SCC15 and OE-PER1-CAL27 cells were significantly increased. All data are from three independent experiments. The data are presented as the mean ± SD values (*n* ≥ 3). *P < 0.05; **P < 0.01; ***P < 0.001; ****P < 0.0001.

Reverse transcription quantitative real-time polymerase chain reaction (RT-qPCR) and western blotting showed that the mRNA and protein expression levels of PER1 in TSCCA, SCC15, and CAL27 OSCC cells were significantly lower than those in HOMEC cells (*P* < 0.001; Fig. 1A, B). From the three OSCC cell lines, TSCCA cells, which had the highest PER1 expression level, were selected to construct stable PER1-knockdown TSCCA cells (RNAi-PER1-TSCCA cells), and cells in the scramble group were used as the negative control. SCC15 and CAL27 cells, which had relatively low PER1 expression levels, were selected to construct SCC15 and CAL27 cells, respectively, with stable overexpression of PER1 (OE-PER1-SCC15 and OE-PER1-CAL27) (Fig. 1C, D). NC-SCC15 and NC-CAL27 cells were used as the corresponding negative controls. Untreated OSCC cells were used as blank controls (blank-TSCCA, blank-SCC15, and blank-CAL27) for the following experiments.

### PER1 regulated glycolysis and proliferation in OSCC cells

**Fig. 2:**
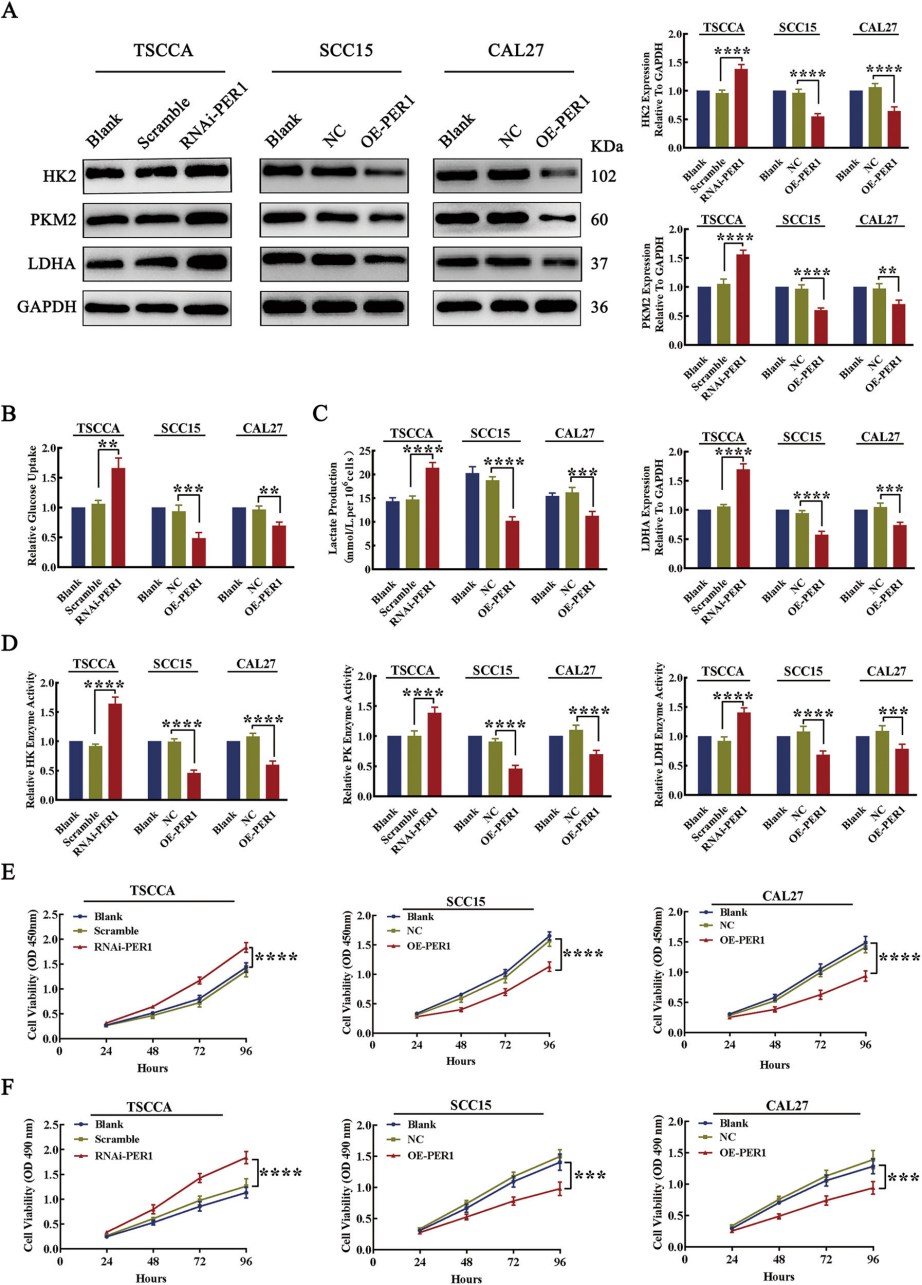
Knockdown of PER1 promoted glycolysis and proliferation in OSCC cells, while overexpression of PER1 inhibited glycolysis and proliferation in OSCC cells. **A** Western blot results showed that the expression levels of HK2, PKM2, and LDHA were significantly increased in RNAi-PER1-TSCCA cells and decreased in OE-PER1-SCC15 and OE-PER1-CAL27 cells. **B**–**D** The results of the glucose uptake, lactate production, and enzyme activity assays showed that glucose uptake, lactate production, and the enzymatic activity of HK, PK, and LDH were significantly increased in RNAi-PER1-TSCCA cells, while in OE-PER1-SCC15 and OE-PER1-CAL27 cells, glucose uptake, lactate production, and the enzymatic activity of HK, PK, and LDH were significantly decreased. **E**, **F** CCK-8 and MTT assays showed that the proliferation of RNAi-PER1-TSCCA cells was significantly increased, while that of OE-PER1-SCC15 and OE-PER1-CAL27 cells was significantly decreased. All data are from three independent experiments. The data are presented as the mean ± SD values (*n* ≥ 3). *P < 0.05; **P < 0.01; ***P < 0.001; ****P < 0.0001.

To explore the effect of PER1 expression on glycolysis in OSCC cells, we evaluated the expression of the key glycolytic proteins HK2, PKM2, and LDHA; glucose uptake; lactate production; and the enzymatic activity of hexokinase (HK), pyruvate kinase (PK), and lactate dehydrogenase (LDH). The results showed that, compared with those in the control group, the expression levels of HK2, PKM2, and LDHA in RNAi-PER1-TSCCA cells was significantly increased (*P* < 0.0001; Fig. 2A); glucose uptake, lactate production and the enzymatic activity of HK, PK, and LDH were also significantly increased (*P* < 0.01; Fig. 2B–D). In contrast, the expression levels of HK2, PKM2, and LDHA in OE-PER1-SCC15 and OE-PER1-CAL27 cells were significantly decreased (*P* < 0.01; Fig. 2A); glucose uptake, lactate production, and the enzymatic activity of HK, PK, and LDH were also significantly decreased (*P* < 0.01; Fig. 2B–D).

To explore the effect of PER1 expression on the proliferation of OSCC cells, cell counting kit-8 (CCK-8) and 3-[4,5-dimethylthiazol-2-yl]-2,5 diphenyl tetrazolium bromide (MTT) assays were used to evaluate cell proliferation. The results showed that, compared to that of the corresponding control cells, the proliferation of RNAi-PER1-TSCCA cells was significantly increased (*P* < 0.0001), while the proliferation of OE-PER1-SCC15 and OE-PER1-CAL27 cells was significantly decreased (*P* < 0.001) (Fig. 2E, F).

**Fig. 3:**
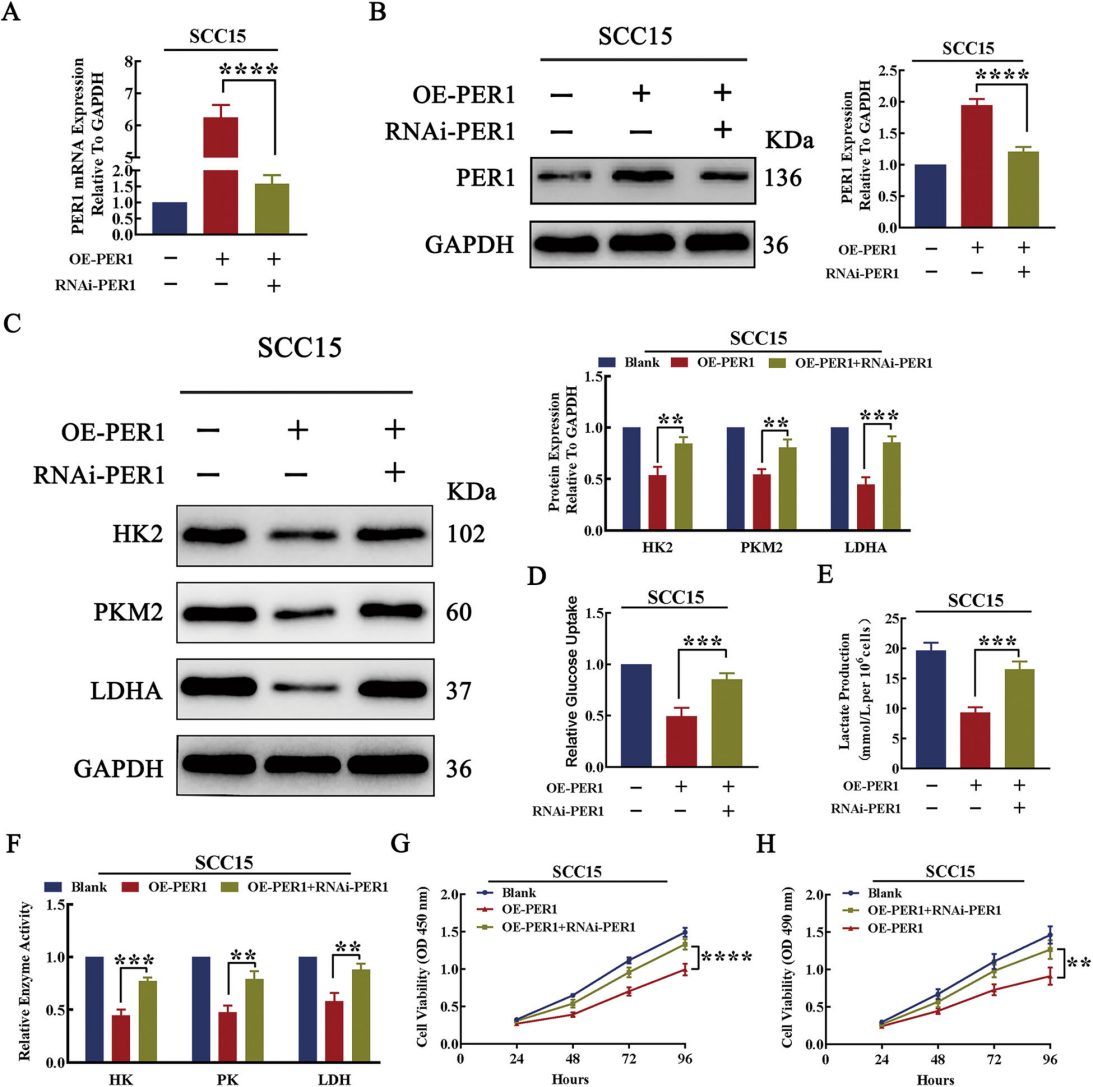
PER1 regulated glycolysis and proliferation in OSCC cells. **A**, **B** The results of RT-qPCR and western blotting showed that the mRNA and protein expression levels of PER1 were significantly decreased after knockdown of PER1 in OE-PER1-SCC15 cells. **C** Western blot results showed that the decrease in the expression of HK2, PKM2, and LDHA was significantly reversed after knockdown of PER1 in OE-PER1-SCC15 cells. **D**–**F** The glucose uptake, lactate production, and enzyme activity assay results showed that the decreases in glucose uptake, lactate production, and enzymatic activity of HK, PK, and LDH were significantly reversed after knockdown of PER1 in OE-PER1-SCC15 cells. **G**, **H** The CCK-8 assay and MTT assay showed that the decrease in proliferation was significantly reversed after knockdown of PER1 in OE-PER1-SCC15 cells. All data are from three independent experiments. The data are presented as the mean ± SD values (*n* ≥ 3). *P < 0.05; **P < 0.01; ***P < 0.001; ****P < 0.0001.

To further demonstrate that PER1 regulates glycolysis and proliferation in OSCC cells, RNAi-PER1 lentivirus was transfected into OE-PER1-SCC15 cells to knock down PER1 for rescue (Fig. 3A, B). The results showed that, after knockdown of PER1 in OE-PER1-SCC15 cells, the PER1 overexpression-mediated decreases in the expression levels of HK2, PKM2, and LDHA; glucose uptake; lactate production; and enzymatic activity of HK, PK, and LDH were significantly reversed (*P* < 0.01; Fig. 3C–F). In addition, the decrease in cell proliferation was significantly reversed (*P* < 0.01; Fig. 3G, H).

These results indicated that PER1 knockdown promoted glycolysis and proliferation in OSCC cells, while PER1 overexpression inhibited glycolysis and proliferation in OSCC cells.

### PER1 regulated glycolysis and proliferation in a PI3K/AKT pathway-dependent manner in OSCC cells

**Fig. 4:**
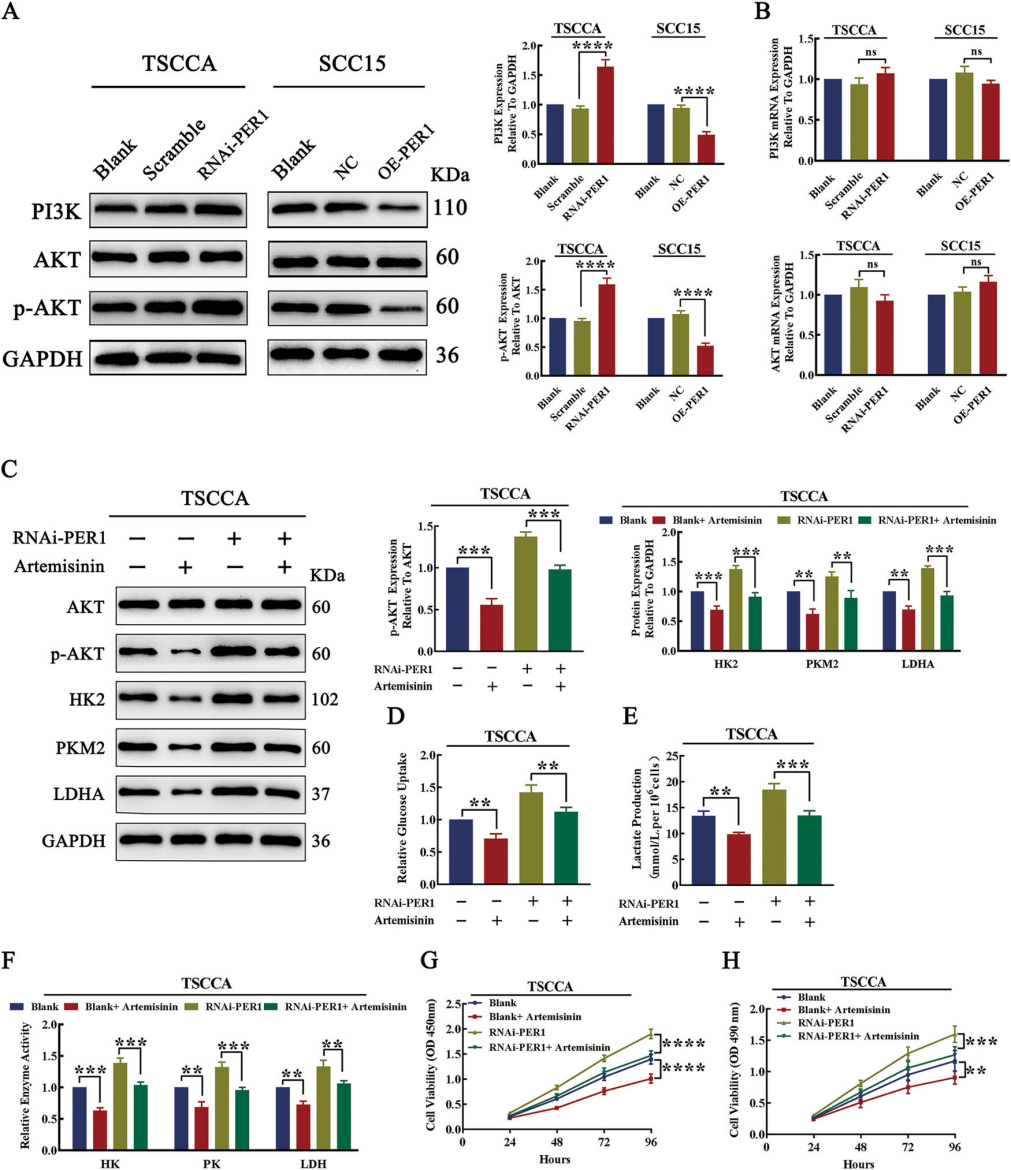
PER1 regulated glycolysis and proliferation in OSCC cells in a PI3K/AKT pathway-dependent manner. **A** Western blot results showed that the levels of PI3K and p-AKT in RNAi-PER1-TSCCA cells were significantly increased, while the levels of PI3K and p-AKT were significantly decreased in OE-PER1-SCC15 cells. **B** RT-qPCR results showed that there were no significant changes in the mRNA levels of PI3K and AKT in RNAi-PER1-TSCCA and OE-PER1-SCC15 cells. **C** Western blot results showed that the increases in the levels of p-AKT, HK2, PKM2, and LDHA were significantly reversed after artemisinin was added to RNAi-PER1-TSCCA cells. **D**–**F** After artemisinin addition, the increases in glucose uptake, lactate production, and the enzymatic activity of HK, PK, and LDH in RNAi-PER1-TSCCA cells were significantly reversed. **G**, **H** The CCK-8 assay and MTT assay showed that the increased proliferation of RNAi-PER1-TSCCA cells was significantly reversed after artemisinin addition. All data are from three independent experiments. The data are presented as the mean ± SD values (*n* ≥ 3). *P < 0.05; **P < 0.01; ***P < 0.001; ****P < 0.0001.

To explore whether PER1 can regulate the PI3K/AKT pathway in OSCC cells, we evaluated the levels of PI3K, AKT, and p-AKT. The western blot results showed that, compared with those in the corresponding control cells, the levels of PI3K and p-AKT in RNAi-PER1-TSCCA cells were significantly increased (*P* < 0.0001), while the levels of PI3K and p-AKT in OE-PER1-SCC15 cells were significantly decreased (*P* < 0.0001) (Fig. 4A). The RT-qPCR results showed no significant changes in PI3K and AKT mRNA levels in RNAi-PER1-TSCCA and OE-PER1-SCC15 cells (*P* > 0.05; Fig. 4B). These results indicated that knockdown of PER1 promoted activation of the PI3K/AKT pathway, and overexpression of PER1 inhibited the PI3K/AKT pathway.

To further explore whether PER1-regulated OSCC glycolysis and proliferation are dependent on the PI3K/AKT pathway, we added the AKT inhibitor artemisinin (HY-B0094, MCE, USA) to RNAi-PER1-TSCCA cells to investigate changes in glycolysis and proliferation. The results showed that adding artemisinin to RNAi-PER1-TSCCA significantly alleviated the PER1 knockdown-mediated increases in the levels of p-AKT, HK2, PKM2, and LDHA; glucose uptake; lactate production; and enzymatic activity of HK, PK, and LDH (*P* < 0.01; Fig. 4C–F). The CCK-8 assay and MTT assay results showed that the increase in cell proliferation was also significantly reversed (*P* < 0.01; Fig. 4G, H). In contrast, we found that when we added the AKT activator SC79 (HY-18749, MCE) to OE-PER1-SCC15 cells, the PER1 overexpression-mediated decreases in the levels of p-AKT, HK2, PKM2, and LDHA; glucose uptake; lactate production; and enzymatic activity of HK, PK, and LDH and cell proliferation were significantly increased (*P* < 0.05; Fig. S1A–F). These results suggested that the regulation of glycolysis and proliferation by PER1 depended on the PI3K/AKT pathway.

### PER1-mediated OSCC cell proliferation depends on the glycolytic pathway

**Fig. 5:**
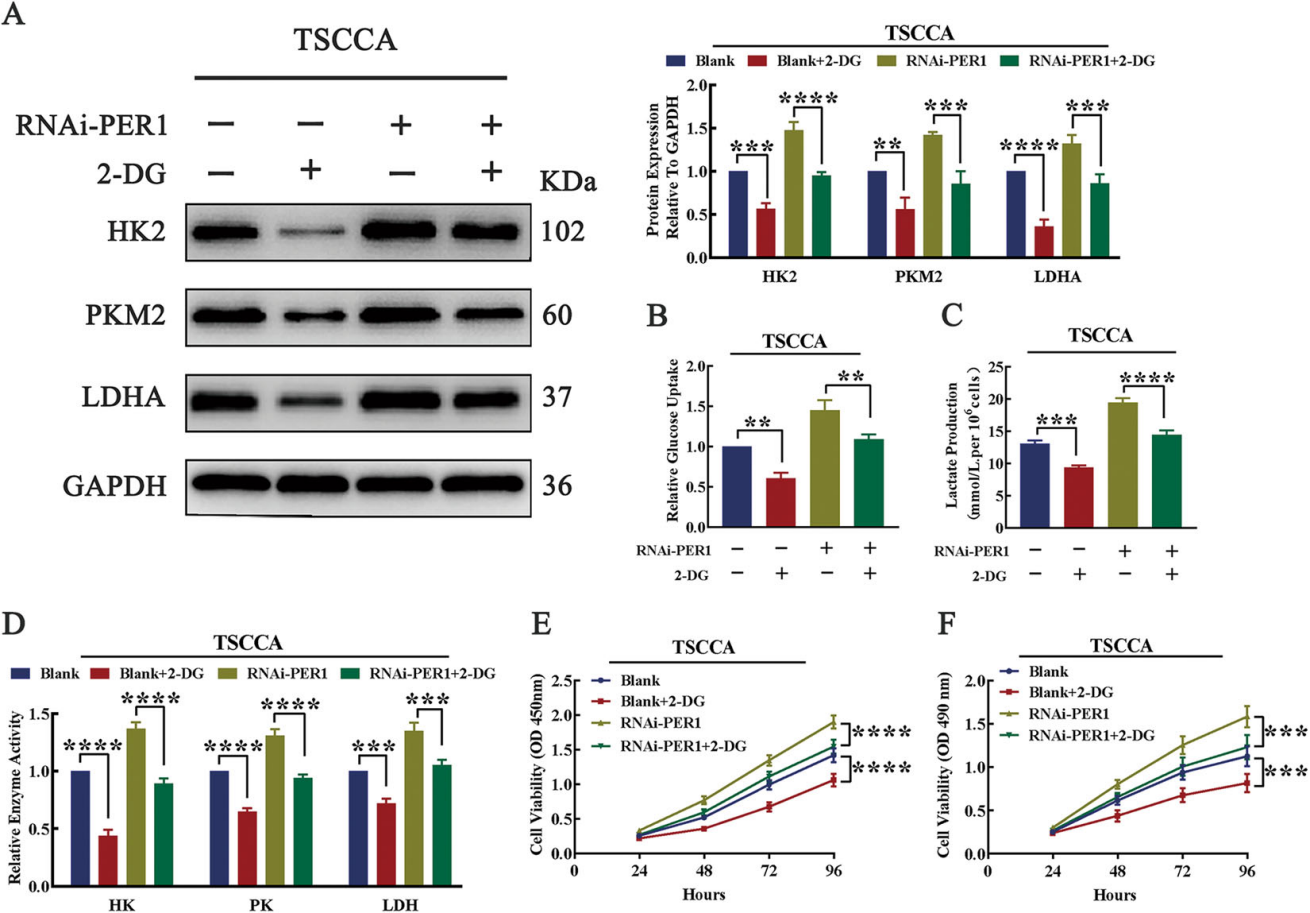
PER1 mediated OSCC cell proliferation through the glycolytic pathway. **A** Western blot results showed that the increase in the expression of HK2, PKM2, and LDHA was significantly reversed after 2-DG was added to RNAi-PER1-TSCCA cells. **B**–**D** After 2-DG was added to RNAi-PER1-TSCCA cells, the increases in glucose uptake, lactate production, and the enzymatic activity of HK, PK, and LDH were significantly reversed. **E**, **F** The CCK-8 assay and MTT assay showed that the increased proliferation of RNAi-PER1-TSCCA cells was significantly reversed after 2-DG addition. All data are from three independent experiments. The data are presented as the mean ± SD values (*n* ≥ 3). *P < 0.05; **P < 0.01; ***P < 0.001; ****P < 0.0001.

To explore whether PER1 mediates the proliferation of OSCC cells through the glycolytic pathway, we added the HK2 inhibitor 2-DG (2-deoxy-d-glucose, HY-13966, MCE) to RNAi-PER1-TSCCA cells to inhibit glycolysis and then evaluated changes in cell proliferation. The results showed that, after 2-DG was added to RNAi-PER1-TSCCA cells, the PER1 knockdown-mediated increases in the expression levels of HK2, PKM2, and LDHA; glucose uptake; lactate production; and enzymatic activity of HK, PK, and LDH were significantly decreased (*P* < 0.01; Fig. 5A–D). In addition, the CCK-8 assay and MTT assay results showed that the increase in cell proliferation was significantly reversed (*P* < 0.001; Fig. 5E, F). These results suggested that PER1 mediated the proliferation of OSCC cells by regulating the glycolytic pathway.

### PER1 regulated the PI3K/AKT pathway, glycolysis, and proliferation in OSCC cells in a manner dependent on the PER1/RACK1/PI3K complex

**Fig. 6:**
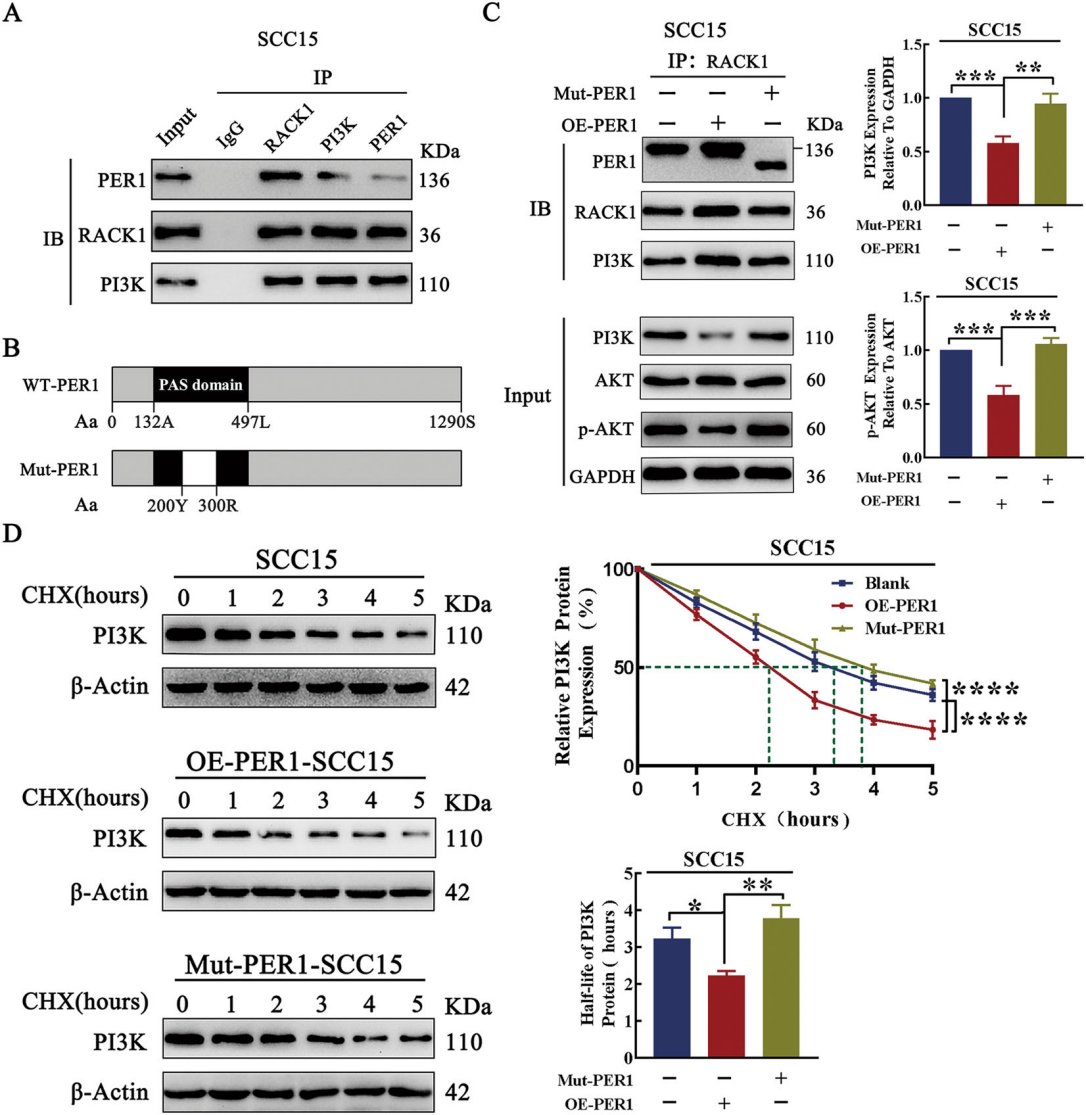
Regulation of the PI3K/AKT pathway via the PER1/RACK1/PI3K complex. **A** The Co-IP results showed that PER1 interacted with RACK1 and PI3K to form the PER1/RACK1/PI3K complex. **B** The amino acid sequences of wild-type and mutant PER1 (the blank box represents the amino acid sequence of the deletion mutant). A stands for alanine, L for leucine, S for serine, Y for tyrosine, and R for arginine. **C** The Co-IP and western blot results showed that, in OE-PER1-SCC15 cells, the abundance of the PER1/RACK1/PI3K complex was significantly increased but the intracellular protein levels of total PI3K and p-AKT were significantly decreased, while in Mut-PER1-SCC15 cells, the increase in the abundance of the PER1/RACK1/PI3K complex and the decreases in the intracellular protein levels of total PI3K and p-AKT were significantly reversed. **D** The CHX chase experiment showed that the half-life of the PI3K protein in OE-PER1-SCC15 cells was significantly decreased, while the decrease in the half-life of the PI3K protein was significantly reversed in Mut-PER1-SCC15 cells. All data are from three independent experiments. The data are presented as the mean ± SD values (*n* ≥ 3). *P < 0.05; **P < 0.01; ***P < 0.001; ****P < 0.0001.

The coimmunoprecipitation (Co-IP) assay results showed that, in OSCC cells, PER1 can interact with RACK1 and PI3K to form the PER1/RACK1/PI3K complex (Fig. 6A). To further explore the regulatory role of the PER1/RACK1/PI3K complex in OSCC cells, we constructed Mut-PER1-SCC15 cells with deletion of the PAS domain (Fig. 6B). The Co-IP and western blot results showed that, compared with that in SCC15 cells, the abundance of the PER1/RACK1/PI3K complex in OE-PER1-SCC15 cells was significantly increased, while the intracellular protein levels of total PI3K and p-AKT were significantly decreased (*P* < 0.001; Fig. 6C). In addition, compared with those in SCC15 cells, lactate production; the enzymatic activity of HK, PK, and LDH; and cell proliferation were significantly decreased in OE-PER1-SCC15 cells (*P* < 0.001; Fig. S2A–D). However, in Mut-PER1-SCC15 cells, the increase in the abundance of the PER1/RACK1/PI3K complex and the decreases in the intracellular protein levels of total PI3K and p-AKT were significantly reversed (*P* < 0.01; Fig. 6C). Moreover, the decreases in lactate production; the enzymatic activity of HK, PK, and LDH; and cell proliferation were significantly rescued (*P* < 0.001; Fig. S2A–D). The cycloheximide (CHX) chase experiment showed that, compared with that in SCC15 cells, the half-life of the PI3K protein in OE-PER1-SCC15 cells was significantly decreased (*P* < 0.05), while the decrease in the half-life of the PI3K protein was significantly reversed in Mut-PER1-SCC15 cells (*P* < 0.01) (Fig. 6D). These results indicated that, in OSCC cells, PER1 promoted the degradation of PI3K through the formation of the PER1/RACK1/PI3K complex, thus regulating the PI3K/AKT pathway, glycolysis, and cell proliferation.

### In vivo tumorigenicity

**Fig. 7:**
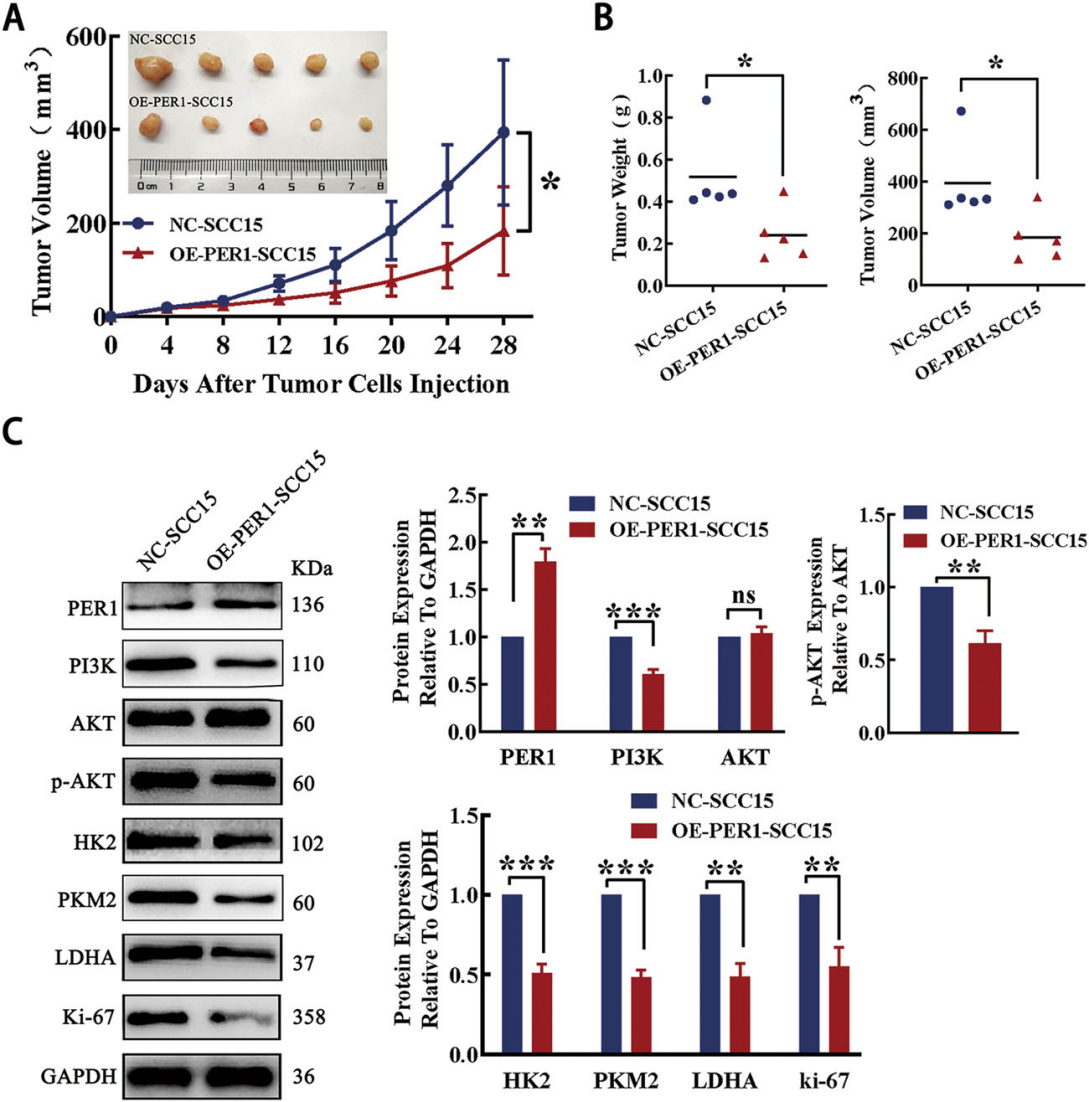
In vivo tumorigenicity. **A** The curve of tumor growth in nude mice showed that the tumor formation rate in the OE-PER1-SCC15 group was significantly lower than that in the NC-SCC15 group. **B** The tumor weights and volumes in the OE-PER1-SCC15 group were significantly lower than those in the NC-SCC15 group. **C** Western blotting showed that the expression of PER1 in the OE-PER1-SCC15 group was significantly higher than that in the NC-SCC15 group, while the levels of PI3K, p-AKT, HK2, PKM2, LDHA, and Ki-67 in the OE-PER1-SCC15 group were significantly lower than those in the NC-SCC15 group. All data are from three independent experiments. The data are presented as the mean ± SD values (*n* ≥ 3). *P < 0.05; **P < 0.01; ***P < 0.001; ****P < 0.0001.

The results of the tumorigenesis assay in nude mice showed that the tumor formation rate, tumor weight and tumor volume in the OE-PER1-SCC15 group were significantly lower than those in the NC-SCC15 group (*P* < 0.01; Fig. 7A, B). Western blotting showed that the expression of PER1 in the OE-PER1-SCC15 group was significantly higher than that in the NC-SCC15 group, while the levels of PI3K, p-AKT, HK2, PKM2, LDHA, and Ki-67 in the OE-PER1-SCC15 group were significantly lower than those in the NC-SCC15 group (*P* < 0.01; Fig. 7C). The above results showed that overexpression of PER1 not only significantly inhibited the growth of OSCC tumors but also significantly inhibited the PI3K/AKT pathway, glycolysis, and proliferation. These results showed that the regulatory mechanism in vivo was consistent with that in vitro.

## Discussion

This study demonstrated that the core circadian clock gene *PER1* was downregulated in OSCC cells. The effects of PER1 were dependent on the formation of the PER1/RACK1/PI3K complex, which negatively regulated the stability of the PI3K protein and activity of the PI3K/AKT pathway; in addition, PER1 regulated glycolysis in a manner dependent on the PI3K/AKT pathway, and the subsequent regulatory effects on cell proliferation were dependent on glycolysis. In vivo tumorigenesis experiments also confirmed that overexpression of PER1 significantly inhibited the growth of OSCC as well as PI3K/AKT pathway activity, glycolysis, and proliferation. We previously found that the expression of PER1 in OSCC tissue was remarkably decreased and was significantly correlated with Tumor, Node, Metastasis clinical stage and that the 5-year survival rate of patients with low expression of PER1 was significantly reduced^15,16^. Zhao et al.^13^ and Liu et al.^14^ reported that the expression of PER1 was low in gastric cancer and NSCLC tissues and that the overall survival time of patients with low expression of PER1 was significantly decreased. This study not only confirmed the previous find that *PER1* has an important suppressive effect on cancers^13,14,29^ but it was importantly found that *PER1* regulates glycolysis and its regulatory mechanism in cancer cells for the first time.

Glucose is the main source of energy for cell growth, and changes in glycolysis levels play a key role in tumor occurrence and development^18^. The occurrence of glycolysis depends on the participation of many important enzymes. HK is the first key rate-limiting enzyme in the glycolytic pathway and is responsible for catalyzing the phosphorylation of glucose to generate glucose-6-phosphate. There are four subtypes of HK, among which the increased expression of HK2 plays an important role in promoting aerobic glycolysis in tumor cells^30^. PK is another key rate-limiting enzyme in the glycolytic pathway; it is responsible for the conversion of phosphoenolpyruvate and ADP into pyruvate and ATP. There are four subtypes of PK, and high expression of PKM2 plays an important role in promoting glycolysis and tumor formation^31,32^. LDH is a key metabolic enzyme that degrades pyruvate into lactate; LDH comprises the LDHA and LDHB subunits, and increased expression of LDHA promotes glycolysis and tumor growth^33,34^. In this study, we established OSCC cells with stable overexpression and knockdown of PER1, and the results showed that OSCC cells overexpressing PER1 exhibited significantly decreased glucose uptake; lactate production; enzymatic activity of HK, PK, and LDH; expression of HK2, PKM2, and LDHA; and proliferation. However, the opposite effects were observed in OSCC cells with PER1 knockdown. Further tumorigenesis experiments in vivo showed that overexpression of PER1 inhibited the growth of OSCC and that the expression of HK2, PKM2, LDHA, and Ki67 in OSCC tissue was significantly decreased. In this study, both the in vitro and in vivo results showed that PER1 had a key negative regulatory effect on glycolysis in OSCC cells.

Current studies have demonstrated that the PI3K/AKT pathway is an important pathway that regulates cell glycolysis and proliferation, thus promoting the occurrence and development of many human tumors, including OSCC^35–37^. We explored whether PER1 regulates glycolysis via the PI3K/AKT pathway and thus affects the occurrence and development of OSCC. To this end, an AKT activator was added to OSCC cells with stable overexpression of PER1, and an AKT inhibitor was added to OSCC cells with stable knockdown of PER1 for functional rescue experiments. The results showed that the regulation of glycolysis by PER1 depended on the PI3K/AKT pathway. To further clarify the relationship between glycolysis and the proliferation of OSCC cells, we added a glycolysis inhibitor to OSCC cells with PER1 knockdown and found that the PER1 knockdown-mediated increase in the proliferation of OSCC cells was significantly alleviated. These results suggest that glycolysis may be the key pathway providing energy for OSCC growth.

The abnormal activation of PI3K/AKT signaling pathway occurs through PI3K promoting AKT phosphorylation (p-AKT)^37–39^. To further explore the mechanism by which PER1 regulates the PI3K/AKT pathway in OSCC cells. We first found that overexpression and knockdown of PER1 resulted in significant decreases and increases, respectively, in the levels of PI3K and p-AKT in OSCC cells, while there was no significant change in PI3K and AKT mRNA levels, which suggests that the regulation of PI3K and AKT by PER1 does not occur at the transcriptional level. Therefore, we explored the mechanism by which PER1 regulates the PI3K/AKT pathway at the level of protein interaction. PER1 belongs to the PAS domain family^40^. Hu et al. reported that PER1 can bind with RACK1 via its PAS domain to form the PER1/RACK1 complex in SCN cells; however, the expression of RACK1 did not change significantly after PER1 knockdown, and the function of the PER1/RACK1 complex is currently unclear^27^. As a scaffold protein, RACK1 can simultaneously interact with multiple protein signaling molecules^27,41^. Zhang et al. reported that RACK1 is highly expressed in OSCC and can upregulate p-AKT expression and promote cell proliferation^42^, but the specific mechanism is unclear. Cao et al. discovered that RACK1 can bind with PI3K to form the RACK1/PI3K complex in human breast cancer cells^28^; however, the changes in PI3K occurring after RACK1 binds with PI3K are currently unclear. In view of the above, we speculate that the PER1/RACK1/PI3K complex may exist in OSCC cells and regulate the PI3K/AKT pathway and cell glycolysis. In this study, we confirmed through Co-IP experiments that PER1 can interact with RACK1 and PI3K to form the PER1/RACK1/PI3K complex in OSCC cells. To clarify the function of the PER1/RACK1/PI3K complex and the mechanism by which it regulates the PI3K/AKT pathway, we further constructed OSCC cells with mutation of PER1 and performed a CHX chase experiment and found that, in OSCC cells, PER1 regulated the stability of the PI3K protein via formation of the PER1/RACK1/PI3K complex while shortened the half-life of PI3K protein, thus regulating the PI3K/AKT pathway, glycolysis, and cell proliferation by promoting PI3K degradation. However, there are still some limitations of this study. First, this study only indicated the existence of the PER1/RACK1/PI3K ternary protein complex in OSCC cells; the specific binding mode of each protein and whether there are other proteins in this complex are still unclear. Second, current studies have shown that the main mechanisms of protein degradation are the ubiquitin-dependent proteasome pathway and autophagy pathway^43^, while the pathway by which the PER1/RACK1/PI3K complex regulated PI3K protein degradation in this study needs further investigation.

In conclusion, in this study, it was found for the first time through in vitro and in vivo experiments that, in OSCC cells, the effects of PER1 depend on the formation of the PER1/RACK1/PI3K complex to regulate PI3K protein stability and the PI3K/AKT pathway; moreover, PER1 regulates glycolysis in a PI3K/AKT pathway-dependent manner, and subsequent regulation of cell proliferation depends on glycolysis, thus affecting the occurrence and development of OSCC. These results suggest that PER1 may be a new therapeutic target in OSCC and that overexpression of PER1 and simultaneous inhibition of glycolysis may become a new effective therapeutic strategy for OSCC.

## Material and methods

### Cell culture and reagents

The human normal oral mucosal cell line HOMEC was purchased from Shanghai Bayley Biotechnology Co., Ltd. (Shanghai, China). The human OSCC cell line TSCCA was purchased from Shanghai Zhongqiao Xinzhou Technology Co., Ltd. (Shanghai, China). SCC15 and CAL27 human OSCC cells were donated by Professor Huang Enyi, Chongqing Key Laboratory of Oral Diseases and Biomedicine. All cell lines were tested for mycoplasma contamination. The cell culture medium consisted of 10% fetal bovine serum (FBS, S711-001 S, Lonsera, Uruguay), 1% penicillin–streptomycin, and 89% Dulbecco’s modified Eagle’s medium (DMEM; Gibco, USA). Cells were cultured in a 37 °C incubator containing 5% CO_2_.

### Construction and packaging of lentiviral vectors for knockdown, overexpression, and mutation of PER1

To construct the PER1-knockdown lentiviral vector, the target sequence for PER1 RNAi (5’-CAGCACCACTAAGCGTAAATG-3’) was designed with the siRNA design software (Invitrogen RNAi Designer Software) based on the human PER1 mRNA sequence (GenBank accession number: NM_002616). Then single-stranded primers for RNAi-PER1 (sense: 5’-CCGGCAGCACCACTAAGCGTAAATGCTCGAGCATTTACGCTTAGTGGTGCTGTTTTTG-3’, antisense: 5’-AATTCAAAAACAGCACCACTAAGCGTAAATGCTCGAGCATTTACGCTTAGTGGTGCTG-3’) were synthesized based on the target sequence for RNAi. Then the PER1-knockdown recombinant plasmid (RNAi-PER1) was constructed by ligating the AgeI/EcoRI double-digested vector GV248 and the double-stranded DNA with T4 DNA ligase. RNAi-PER1 lentivirus was obtained by mixing the RNAi-PER1 plasmid with Lipofectamine 2000 and adding the mixture to 293T cell culture medium for culture.

To construct the PER1-overexpression lentiviral vector, PCR amplification primers specific for the *PER1* gene (forward primer sequence: 5’-GCCAACCAGGAATACTACCAGC-3’, reverse primer sequence: 5’-GTGTGTACTCAGACGTGATGTG-3’) were designed and synthesized based on the sequence of human PER1 mRNA. The PER1 sequence was amplified by PCR, and the PCR products were recovered and ligated into the GV492 vector digested with AgeI endonuclease to construct the recombinant plasmid for PER1 overexpression (OE-PER1). The OE-PER1 plasmid was mixed with Lipofectamine 2000 and transfected into 293T cells to obtain OE-PER1 lentivirus.

To construct the PER1 deletion mutant lentivirus vector, two pairs of primers, A/B and C/D (A: 5’-CCGCTCGAGCCGATGACACCGATGCC-3’, B: 5’-TTAGGCGGAATGGCTGGCTGCTGGTAGTATTCCT-3’, C: 5’-AGGAATACTACCAGCAGCCAGCCATTCCGCCTAA-3’, D: 5’-CGCGGATCCCCCGAGACTCAATAAAA-3’), were designed to amplify the regions upstream and downstream, respectively, of bp 601–900 with Primer Premier 5.0 (Premier Software, CA) based on the DNA sequence of PER1. Using the wild-type PER1 DNA sequence as a template, the homologous base sequences upstream and downstream of the PER1 DNA fragment (bp 601–900) to be deleted were amplified with the abovementioned pairs of primers as indicated. The PCR products of the upstream and downstream homologous base sequences were used as templates and the primer pair A/D was used for fusion PCR to obtain the homologous base sequence with a deletion mutation in the PER1 PAS domain. The recovered products of the purified homologous base sequences and the GV143 vector were double digested with XhoI/BamHI and were then ligated with T4 DNA ligase to construct the recombinant plasmid with a PER1 deletion mutation (Mut-PER1). The Mut-PER1 plasmid was mixed with Lipofectamine 2000 and transfected into 293T cells to obtain Mut-PER1 lentivirus.

### Transfection of PER1-knockdown, PER1-overexpression, and PER1-mutation lentivirus

TSCCA, SCC15, and CAL27 OSCC cells in logarithmic growth phase were separately prepared into cell suspensions with a density of 5 × 10^4^ cells/ml in DMEM and were then inoculated in a 6-well plate (2 ml per well). The plate was then placed in an incubator (37 °C, 5% CO_2_) overnight, and 40 µl of HiTransG P infection-enhancing solution (1×) was then added. Then, assuming a multiplicity of infection (MOI) of 50, the corresponding volume of PER1-knockdown, PER1-overexpression, or PER1-mutation lentivirus was added to the infection reagent based on the following equation: virus volume = (MOI × cell number)/virus titer. Seventy-two hours after infection, the infection efficiency was evaluated under a fluorescence microscope (Eclipse Ti, NIKON, Japan). Then cells were selected in medium containing 2 μg/ml puromycin, 10% FBS, and 1% penicillin–streptomycin. After 7 days, the concentration of puromycin in the medium was adjusted to 1 μg/ml for routine subculture. TSCCA cells with stable knockdown of PER1 (RNAi-PER1-TSCCA), SCC15 and CAL27 cells with stable overexpression of PER1 (OE-PER1-SCC15 and OE-PER1-CAL27), and SCC15 cells with stable deletion mutation of PER1 (Mut-PER1-SCC15) were thus obtained. TSCCA cells were infected with lentivirus expressing a scrambled plasmid (scramble sequence: TTCTCCGAACGTGTCACGT) without the PER1 gene sequence as a negative control group. SCC15 and CAL27 cells were infected with lentivirus expressing empty vector without the PER1 gene sequence as negative control groups (the NC-SCC15 group and NC-CAL27 group).

### CCK-8 assay

CCK-8 (ck04, Dojindo, China) was used to evaluate cell proliferation. A 100-μl cell suspension with a density of 1 × 10^4^ cells/ml was added to each well of a 96-well plate, and triplicate wells were established for each group. Then the plate was placed in an incubator (37 °C, 5% CO_2_) overnight. At 24, 48, 72, and 96 h, 100 μl of medium containing 10% CCK-8 reagent was added to each well. After incubation for 2 h in an incubator, the optical density (OD) value at 450 nm was measured in an enzyme labeling instrument (BioTek, USA). A cell growth curve was drawn with time as the abscissa and the OD value as the ordinate. The experiment was repeated three times.

### MTT assay

Cell proliferation was evaluated with an MTT Kit (11465007001, Roche, Switzerland). A cell suspension at a density of 1 × 10^4^ cells/ml was inoculated into a 96-well plate (100 μl per well), with triplicate wells established for each group. Then the plate was placed in an incubator (37 °C, 5% CO_2_), and after 24, 48, 72, and 96 h, 20 μl of MTT solution was added to the medium. After further incubation for 4 h in the incubator, 150 μl of dimethyl sulfoxide was added to each well after the medium was removed, and the OD value at 490 nm was measured in an enzyme labeling instrument. Finally, a cell viability curve was drawn with time as the abscissa and OD value as the ordinate. The experiment was repeated three times.

### RT-qPCR assay

Total RNA was extracted according to the instructions for RNAiso Plus (9180, Takara, Japan). The purity and concentration of total RNA were determined by microspectrophotometry (NanoPhotometer, Implen, Germany). Total RNA (1 μg) was reverse transcribed into cDNA in a 20-µl reaction volume with a PrimeScript RT Reagent Kit with gDNA Eraser (RR047A, Takara). The reaction conditions were as follows: 37 °C for 15 min, 85 °C for 5 s, and holding at 4 °C. PCR primers specific for PER1, PI3K, AKT, and glyceraldehyde 3-phosphate dehydrogenase (GAPDH) were designed with Oligo 7.0 (Primer Analysis Software, Oligo; Table S1). PCR was performed according to the instructions for SYBR Premix Ex Taq (RR820A, Takara) in a reaction system of 10 µl. PCR amplification was carried out in a C-1000^TM^ thermal cycler. The reaction conditions were as follows: predenaturation at 95 °C for 1.5 min, denaturation at 95 °C for 10 s, and annealing at 60 °C for 30 s, with 40 amplification cycles. Each sample was divided into three wells. The relative mRNA expression level of the target gene was calculated based on the formula 2^−ΔΔCt^.

### Western blotting

After cells were collected, RIPA lysis buffer (P0013B, Beyotime, China) containing 1% protease inhibitor and 2% phosphatase inhibitor was added, and the cells were lysed in an ultrasonicator (10 s × 3 times at intervals of 10 s) and placed on ice for 30 min. After centrifugation for 15 min (4 °C, 12,000 rpm), the supernatant was collected. A BCA protein quantitation kit (P0010, Beyotime) was used to determine the protein concentration. Total protein (30–50 g) was separated by 8–12% sodium dodecyl sulfate (SDS)-polyacrylamide gel electrophoresis, and proteins were transferred to polyvinylidene difluoride (PVDF) membranes with a 0.45-µm pore diameter by the wet transfer method. PVDF membranes were blocked in buffer containing 5% skim milk powder or 5% bovine serum albumin for 1 h at room temperature. Then they were incubated with primary antibodies (Table S2) against PER1, PI3K, AKT, p-AKT, HK2, PKM2, LDHA, and GAPDH (1:1000) at 4 °C overnight. PVDF membranes were washed three times with TBST for 10 min each. Then PVDF membranes were incubated with a horseradish peroxidase-conjugated rabbit secondary antibody (1:5000) at room temperature for 1 h. PVDF membranes were then washed three times with TBST for 10 min each. A hypersensitive chemiluminescent solution (P0018S, Beyotime) was prepared at a ratio of 1:1. Appropriate amounts of the hypersensitive chemiluminescent solution were evenly dripped on the PVDF membranes, which were then developed in an ECL Advance western blot detection system (Bio-Rad, USA) to acquire images. AKT was used as the normalization control for p-AKT, and GAPDH was used as the normalization control for other proteins. ImageJ 5.0 software (ImageJ bundled with 64-bit Java 1.8.0_ 112) was used for grayscale analysis of the protein bands.

### Determination of glucose uptake, lactate production, and enzymatic activity

OSCC cells in logarithmic growth phase were digested with 0.25% trypsin and centrifuged (rt, 1000 rpm) for 10 min. Then the cells were centrifuged in phosphate-buffered saline (PBS) buffer (rt, 1000 rpm) for 5 min and washed twice. Then 500 μl of PBS was added to the collected cells, and the cells were suspended and lysed in an ultrasonicator (power, 300 W; 5 s × 3 times with 4 intervals of 30 s each; in an ice-water bath). A BCA protein quantitation kit was used to determine the protein concentration in each group.

#### Determination of glucose uptake

Glucose uptake was measured with a glucose determination kit (Rongsheng Biology, China). According to the instructions of the kit, the reaction solution in the sample tube, calibration tube, and blank tube was prepared based on the kit components, evenly mixed and placed in a water bath at 37 °C for 10 min. Then the reaction solution was extracted and added to a 96-well plate (200 µl per well); triplicate wells were established for each group. The OD of each well at 550 nm was measured in an enzyme labeling instrument. Glucose uptake (mmol/l) = sample tube OD value/(calibration tube OD value × calibration tube concentration). The experiment was repeated three times.

#### Determination of lactate production

A lactic acid kit (A019-2-1, Nanjing Jiancheng, China) was used to measure lactate production. The enzyme working solution and chromogenic agent were prepared based on the instructions of the kit, and 1 ml of enzyme working solution and 0.2 ml of chromogenic reagent were then added successively to 0.02 ml of double distilled water (blank tube), 0.02 ml of 3 mmol/l standard (standard tube), or 0.02 ml of the sample to be tested (determination tube). After mixing, the samples were reacted in a water bath at 37 °C for 10 min. Finally, 2 ml of termination solution was added to each tube. After mixing, the reaction solution was extracted and added to a 96-well plate (200 µl per well). Triplicate wells were established for each group. The OD value of each well at 530 nm was measured in an enzyme labeling instrument. Lactate production (mmol/l) = (determination tube OD value − blank tube OD value)/(standard tube OD value − blank tube OD value) × (standard concentration/sample protein concentration). The experiment was repeated three times.

#### Determination of HK activity

HK activity was measured with a HK assay kit (A077-1, Nanjing Jiancheng). The working solution was prepared according to the proportions defined in the kit instructions, and the prepared working solution was put into a water bath at 37 °C for 10 min. Then 0.96 ml of preheated working solution was added to a test tube containing 50 µl of the sample to be tested. After rapid mixing, the reaction solution was extracted and added to a 96-well plate (200 µl per well), with triplicate wells established for each group. The OD value of each well at 340 nm was measured with an enzyme labeling instrument and was recorded as A1. Then the remaining reaction solution in the test tube was placed in a water bath at 37 °C for precisely 2 min, and the reaction solution was then quickly extracted and added to a 96-well plate (200 µl per well), with triplicate wells for each group. The OD value of each well at 340 nm was measured with an enzyme labeling instrument and was recorded as A2. HK activity (U/g protein) = ΔA/(molar extinction coefficient)/reaction time/(colorimetric light path) × (sample dilution ratio in reaction system/sample protein concentration), where ΔA = A2 − A1. The experiment was repeated three times.

#### Determination of pyruvate kinase (PK) activity

Pyruvate kinase activity was measured with a pyruvate kinase assay kit (A076-1, Nanjing Jiancheng). The working solution was prepared according to the kit instructions based on the components. After mixing the prepared working solution and placing it in a water bath at 37 °C for 10 min, 20 µl of the sample to be tested was added to the preheated working solution. After rapid mixing, the reaction solution was quickly extracted and added to a 96-well plate (200 µl per well), with triplicate wells for each group. The OD value of each well at 340 nm was measured with an enzyme labeling instrument and was recorded as A1. After that, the remaining reaction solution in the test tube was placed in a water bath at 37 °C for precisely 15 min, and the reaction solution was then quickly extracted and added to a 96-well plate (200 µl per well). Triplicate wells were established for each group. The OD value of each well at 340 nm was measured with an enzyme labeling instrument and was recorded as A2. PK activity (U/g protein) = ΔA/(molar extinction coefficient)/reaction time/colorimetric light path × total volume of reaction solution/sampling volume/sample protein concentration, where ΔA = A1 − A2. The experiment was repeated three times.

#### Determination of LDH kinase activity

A LDH kinase assay kit (A020-1, Nanjing Jiancheng) was used to evaluate LDH kinase activity. The reaction solution in the blank tube, standard tube, measurement tube, and control tube were prepared according to the instructions of the kit based on the components and were evenly mixed, and the reaction solution was placed in a water bath at 37 °C for 15 min. Then 0.25 ml of 2,4-nitrophenylhydrazine was added to the reaction solution; after mixing the solution, it was placed in a water bath at 37 °C for 15 min, and 2.5 ml of 0.4 mol/l NaOH solution was finally added to the reaction solution. After mixing, the reaction solution was incubated at room temperature for 3 min. Then the reaction solution was extracted and added to a 96-well plate (200 µl per well), with triplicate wells for each group. The OD value of each well at 340 nm was measured with an enzyme labeling instrument. LDH activity (g protein/ml) = (measured OD value − control OD value)/(standard OD value − blank OD value) × (standard concentration/sample protein concentration). The experiment was repeated three times.

### Coimmunoprecipitation

One microgram of anti-PER1, anti-RACK1, or anti-PI3K antibodies was added separately to SCC15 cell lysates and incubated at 4 °C overnight. An appropriate amount of RIPA lysis buffer was added to 10 µl of protein A/G beads, and centrifuged (4 °C, 3000 rpm) 3 times for 3 min each. The pretreated 10 µl beads were added to the SCC15 cell lysate incubated overnight with the antibody, incubated at 4 °C for 3 h, and centrifuged (4 °C, 3000 rpm) for 3 min; the supernatant was then discarded. After that, the beads were washed with RIPA lysis buffer 3 times for 30 s each and were then centrifuged (4 °C, 3000 rpm) for 30 s. The supernatant was aspirated, and the beads and an appropriate amount of protein lysate were retained. Then 20 µl of 2 × SDS loading buffer was added to the retained beads and protein lysate, boiled at 100 °C for 5 min, and analyzed by western blotting.

### CHX chase experiment

SCC15, OE-PER1-SCC15, and Mut-PER1-SCC15 cells were inoculated into 6 different 6-cm dishes, respectively. After the cells adhered to the wall, the old medium was discarded, and 2 ml of DMEM containing the same dose of 100 μg/ml CHX (Sigma-Aldrich, USA) was added to each dish of cells. Then total protein was extracted at time points of 0, 1, 2, 3, 4, and 5 h after the addition of CHX and analyzed by western blotting.

### In vivo tumorigenicity assay

Ten SPF-grade female BALB/c nu/nu mice (weight, 18–22 g; age, 4–6 weeks) were randomly divided into the OE-PER1-SCC15 group (experimental group) and NC-SCC15 group (control group) with the random number table method, with 5 mice in each group. OE-PER1-SCC15 and NC-SCC15 cell suspensions (0.2 ml) at a concentration of 5 × 10^6^ cells/ml were injected subcutaneously into the left dorsal region of nude mice in the experimental group and the control group, respectively. Tumor growth was observed and recorded every 3 days for 4 weeks. After tumor formation was obvious, the nude mice were sacrificed by cervical dislocation. The subcutaneous tumors were separated, and the tumors were weighed. The maximum long diameter (*a*) and the minimum short diameter (*b*) of the tumor were measured with a Vernier caliper, and the tumor volume (*V*) was calculated as 0.5 × *a* × *b*^2^. The levels of PER1, PI3K, AKT, p-AKT, HK2, PKM2, LDHA, and Ki-67 were measured by western blotting. This study was approved by the Ethics Committee of the First Affiliated Hospital of Chongqing Medical University (Approval number: 2018-102).

### Statistical analysis

GraphPad Prism 8.0 (GraphPad Software, La Jolla, CA) and SPSS 23 (IBM, SPSS, Chicago, IL, USA) were used for data processing and statistical analysis. The data for each set of three independent replicates are presented as the mean ± SD values. Student’s *t* test was used for comparisons between two independent sample groups, one-way analysis of variance (ANOVA) was used for single-factor comparisons among multiple groups, and two-way ANOVA was used for two-factor comparisons among multiple groups. *P* < 0.05 was considered statistically significant.

## Supplementary information

Table S1.

Table S2

Supplementary Figure legends

Fig. S1

Fig. S2
