# Supplementary material for: PER1 suppresses glycolysis and cell proliferation in oral squamous cell carcinoma via the PER1/RACK1/PI3K signaling complex
Source: Cell Death Dis. 2021 Mar 15;12(3):276. doi: 10.1038/s41419-021-03563-5 (PMC7960720; doi:10.1038/s41419-021-03563-5)
Supplement: Supplementary file 1 — Table S1. [file 41419_2021_3563_MOESM1_ESM.docx]

**Table S1.** Primer sequences for RT-qPCR

| **Gene Forward primer sequence (5' to 3') Reverse primer sequence (5' to 3')** | | |
| --- | --- | --- |
| PER1  PI3K  AKT  GAPDH | GCCAACCAGGAATACTACCAGC  CCACGACCATCATCAGGTGAA  AGCGACGTGGCTATTGTGAAG  TCAAGAAGGTGGTGAAGCAGG | GTGTGTACTCAGACGTGATGTG  CCTCACGGAGGCATTCTAAAGT  GCCATCATTCTTGAGGAGGAAGT  AGCGTCAAAGGTGGAGGAGTG |
