# Supplementary material for: PER1 suppresses glycolysis and cell proliferation in oral squamous cell carcinoma via the PER1/RACK1/PI3K signaling complex
Source: Cell Death Dis. 2021 Mar 15;12(3):276. doi: 10.1038/s41419-021-03563-5 (PMC7960720; doi:10.1038/s41419-021-03563-5)
Supplement: Supplementary file 2 — Table S2 [file 41419_2021_3563_MOESM2_ESM.docx]

**Table S2.** Antibody information for western blotting

| **Antibody** | **Catalog NO.** | **brand** | **city** | **Country** |
| --- | --- | --- | --- | --- |
| PER1 | GTX128974 | GeneTex | California | America |
| PI3K | #4249 | CST | Massachusetts | America |
| AKT | #4691 | CST | Massachusetts | America |
| p-AKT(ser473) | #4060 | CST | Massachusetts | America |
| HK2 | #2867 | CST | Massachusetts | America |
| PKM2 | #4053 | CST | Massachusetts | America |
| LDHA | #3582 | CST | Massachusetts | America |
| RACK1 | #5432 | CST | Massachusetts | America |
| Ki-67 | ab16667 | abcam | Cambridge | Britain |
| β-Actin | #4970 | CST | Massachusetts | America |
| GAPDH | 10494-1-AP | Proteintech | Chicago | America |
| HRP-conjugated |  |  |  |  |
| Affinipure Goat | SA00001-2 | Proteintech | Chicago | America |
| Anti-Rabbit IgG  (H+L) |  |  |  |  |
