# Supplementary material for: PER1 suppresses glycolysis and cell proliferation in oral squamous cell carcinoma via the PER1/RACK1/PI3K signaling complex
Source: Cell Death Dis. 2021 Mar 15;12(3):276. doi: 10.1038/s41419-021-03563-5 (PMC7960720; doi:10.1038/s41419-021-03563-5)
Supplement: Supplementary file 3 — Supplementary Figure legends [file 41419_2021_3563_MOESM3_ESM.docx]

**Supplementary Figure legends**

**Fig. S1 PER1 regulated glycolysis and proliferation in OSCC cells in a PI3K/AKT pathway-dependent manner.** A, Western blotting showed that the decreases in the levels of p-AKT, HK2, PKM2 and LDHA was significantly reversed in OE-PER1-SCC15 cells treated with SC79. B, C, D, After SC79 was added to OE-PER1-SCC15 cells, the decreases in glucose uptake, lactate production and the enzymatic activity of HK, PK and LDH were significantly reversed. E, F, The CCK-8 assay and MTT assay showed that the decreased proliferation of OE-PER1-SCC15 cells was significantly reversed after SC79 addition. All data are from three independent experiments. The data are presented as the mean ± SD values (n ≥ 3). *, *P* < 0.05; **, *P* < 0.01; ***, *P* < 0.001; ****, *P* < 0.0001.

**Fig. S2 Regulation of glycolysis and proliferation via the PER1/RACK1/PI3K complex.** A, B, The results of lactate production and enzyme activity assays showed that lactate production and the enzymatic activity of HK, PK and LDH were significantly decreased in OE-PER1-SCC15 cells, while the decreases in lactate production and the enzymatic activity of HK, PK and LDH were significantly reversed in Mut-PER1-SCC15 cells. C, D, The CCK-8 assay and MTT assay showed that the proliferation of OE-PER1-SCC15 cells decreased significantly, while the decreased proliferation was significantly reversed in Mut-PER1-SCC15 cells. All data are from three independent experiments. The data are presented as the mean ± SD values (n ≥ 3). *, *P* < 0.05; **, *P* < 0.01; ***, *P* < 0.001; ****, *P* < 0.0001.
