# Supplementary figures and images for: PER1 suppresses glycolysis and cell proliferation in oral squamous cell carcinoma via the PER1/RACK1/PI3K signaling complex
Source: Cell Death Dis. 2021 Mar 15;12(3):276. doi: 10.1038/s41419-021-03563-5 (PMC7960720; doi:10.1038/s41419-021-03563-5)

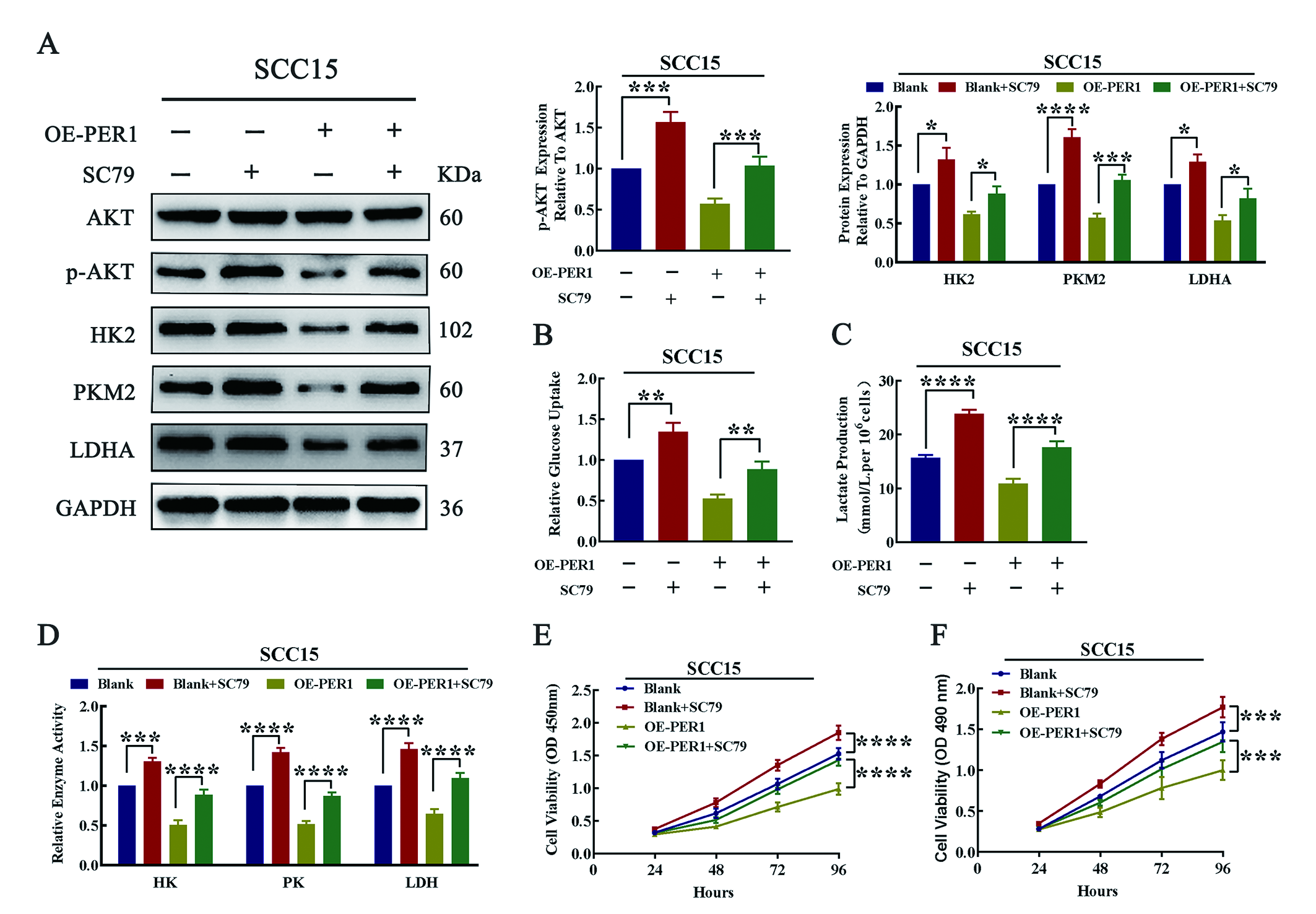

Supplement: Supplementary file 4 — Fig. S1 [file 41419_2021_3563_MOESM4_ESM.tif]

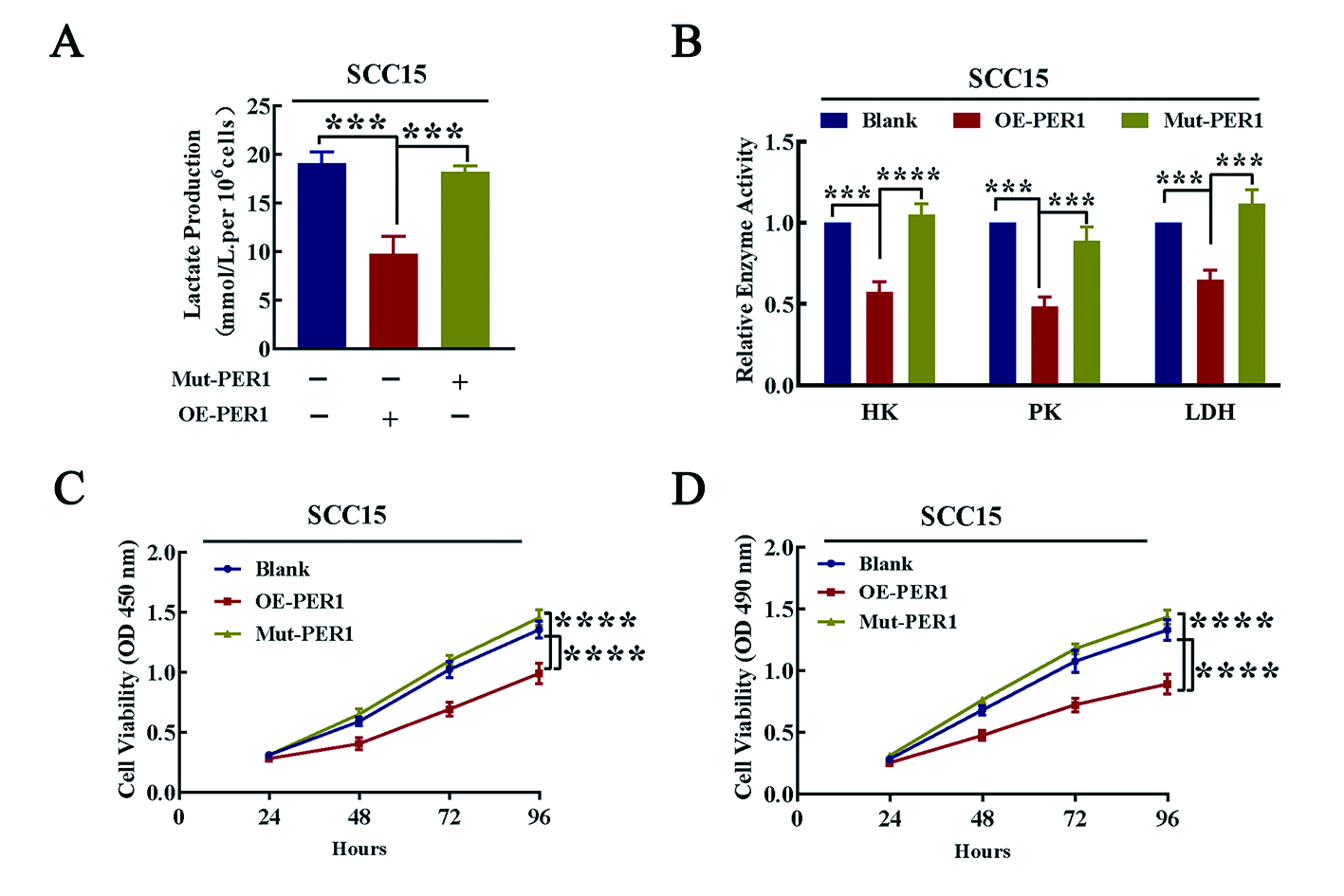

Supplement: Supplementary file 5 — Fig. S2 [file 41419_2021_3563_MOESM5_ESM.tif]
